# Treatment of Distal Femoral Malignant Mesenchymal Tumor With Extensor Mechanism Transfer: A Case Report and Surgical Technique

**DOI:** 10.7759/cureus.75303

**Published:** 2024-12-07

**Authors:** Arın Celayir, Burak Ozturk, Şeyhmus Kavak, Mahmut K Ozsahin, Huseyin Botanlioglu

**Affiliations:** 1 Orthopaedics and Traumatology, Cerrahpaşa Faculty of Medicine, Istanbul University-Cerrahpaşa, Istanbul, TUR

**Keywords:** extensor mechanism transfer, gracilis, malignant mesenchymal tumor, medial parapatellar approach, wide resection

## Abstract

Malignant mesenchymal tumors are a diverse group of aggressive cancers originating from mesenchymal cells in connective tissues such as bone, muscle, cartilage, and fat. These tumors often invade surrounding tissues and metastasize to distant organs, posing significant treatment challenges. Among them, malignant mesenchymal tumors located in the distal femur are particularly rare, with limited reports detailing effective surgical and functional reconstruction strategies following wide resection. This case report focuses on the treatment of a distal femoral malignant mesenchymal tumor managed at our clinic, where wide resection was followed by reconstruction using a tumor resection prosthesis and extensor mechanism transfer. The report highlights the innovative surgical technique employed to restore extensor function while maintaining structural integrity, addressing a critical gap in the literature. By sharing insights into this approach, this report aims to contribute to the growing body of knowledge on limb salvage surgery and improve outcomes for patients with similar complex conditions.

## Introduction

Malignant mesenchymal tumors are diverse and aggressive cancers arising from connective tissues, requiring tailored treatment strategies that often combine surgery, chemotherapy, and radiation therapy [1]. These therapies aim to remove the tumor, target cancer cells, and destroy residual disease, respectively, depending on the tumor's type, location, and stage, as well as the patient's overall health [2,3]. While their exact causes remain unclear, these tumors are linked to genetic mutations, chromosomal abnormalities, and exposure to carcinogens like radiation or certain chemicals. Genetic predispositions, prior radiation therapy, or injuries to the affected region may also increase susceptibility [4].

These tumors commonly harbor mutations in key genes involved in cell growth regulation, such as TP53, RB1, and PTEN, as well as alterations in oncogenes like MYC and KRAS. Chromosomal rearrangements, deletions, and amplifications are also prevalent, disrupting critical signaling pathways that control cell proliferation and survival. Furthermore, epigenetic modifications, including DNA methylation and histone modifications, contribute to the dysregulation of gene expression in these tumors [5].

Surgical treatment plays a pivotal role in managing malignant mesenchymal tumors, aiming to remove the tumor while preserving surrounding healthy tissue. Surgeons use various techniques, such as excision, to achieve complete tumor removal whenever possible. The extent of surgery depends on factors like tumor size, location, and its relationship with adjacent structures. In some cases, surgical resection may be curative, particularly for localized tumors. However, due to the aggressive nature of these cancers and their potential to spread, adjuvant therapies like chemotherapy or radiation may be necessary to target residual cancer cells or prevent recurrence after surgery [6].

This case report discusses the method of repairing the extensor mechanism, which was performed on a patient with a malignant mesenchymal tumor located in the distal left femur. The patient had undergone extensive resection and received reconstruction using a tumor resection prosthesis at our clinic.

## Case presentation

**Figure 1:**
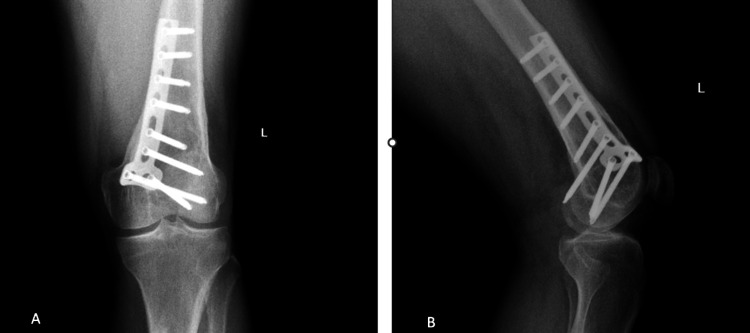
X- ray images of the patient at the time of hospital admission. (A) Left knee anterior-posterior view. (B) Left knee lateral view.

A 44-year-old male patient presented to our clinic with complaints of pain and limited movement in the left knee. Before presenting to us, the patient had sustained a distal femur fracture following a fall and underwent surgery at an external center. Incidentally, a lesion was observed during the procedure, prompting a biopsy, which was reported as a malignant mesenchymal tumor. Whole-body positron emission tomography (PET) imaging performed at our clinic revealed no evidence of distant metastases. Informed consent was obtained from the patient before any procedures were performed. A direct X-ray was taken (Figure 1).

**Figure 2:**
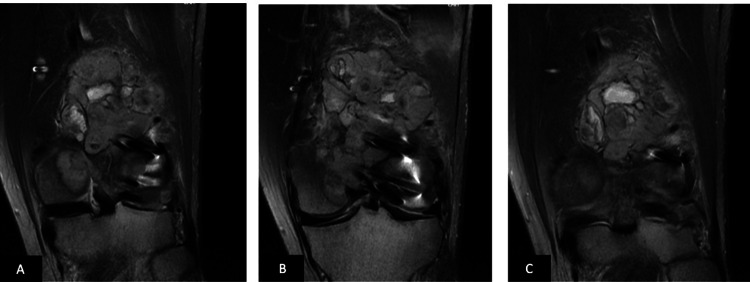
Magnetic resonance images of the patient. (A-B-C) Coronal views of the patient.

**Figure 3:**
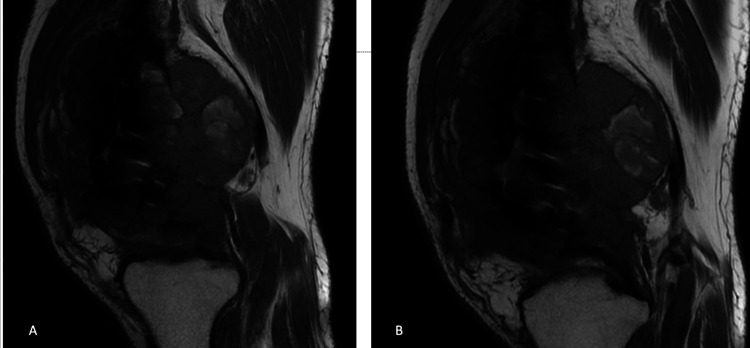
Magnetic resonance images of the patient. (A-B) Sagittal views of the patient.

**Figure 4:**
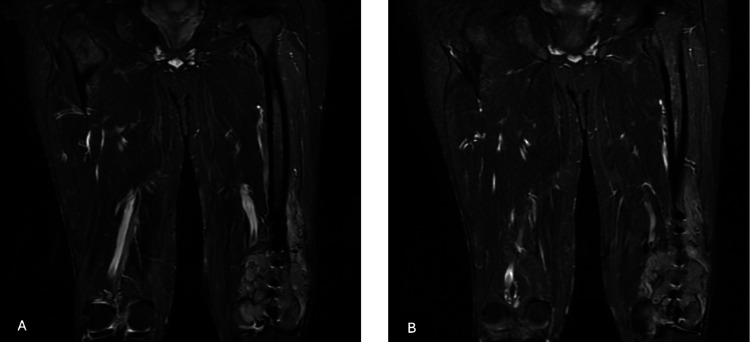
Magnetic resonance images of the patient. (A-B) Coronal views of the patient showing no skip metastasis.

It was observed that the patient had suffered a fracture in the distal left femur three months prior to a fall, for which open reduction and internal fixation had been performed externally. Subsequently, the patient underwent contrast-enhanced MRI examination of the bilateral lower extremities, and skip metastasis was ruled out (Figures 2-4).

**Figure 5:**
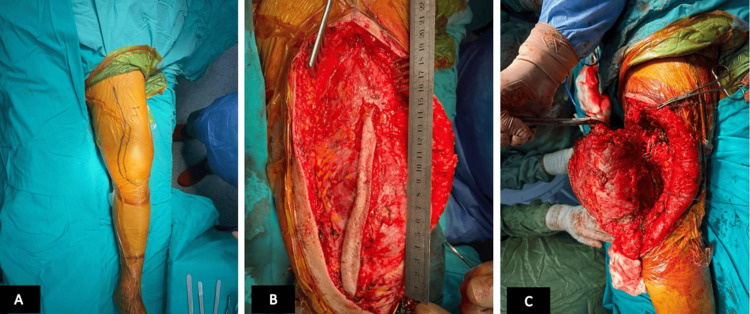
Peroperative images of the patient. (A) Before the surgery. (B) Longitudinal view of the lesion before removal. (C) Side view of the lesion before removal.

The MRI revealed an expansile mass measuring 8x7x7 cm. Following a detailed evaluation at the tumor board, the patient was indicated for wide resection and reconstruction with a tumor resection prosthesis, as well as adjuvant chemotherapy. In the preoperative planning, a 14 cm bone incision and a 16 cm soft tissue resection margin were defined from the distal to the proximal femur. As the implants located in the distal femur were included within the wide resection margin, a separate implant extraction procedure was not performed (Figure 5).

**Figure 6:**
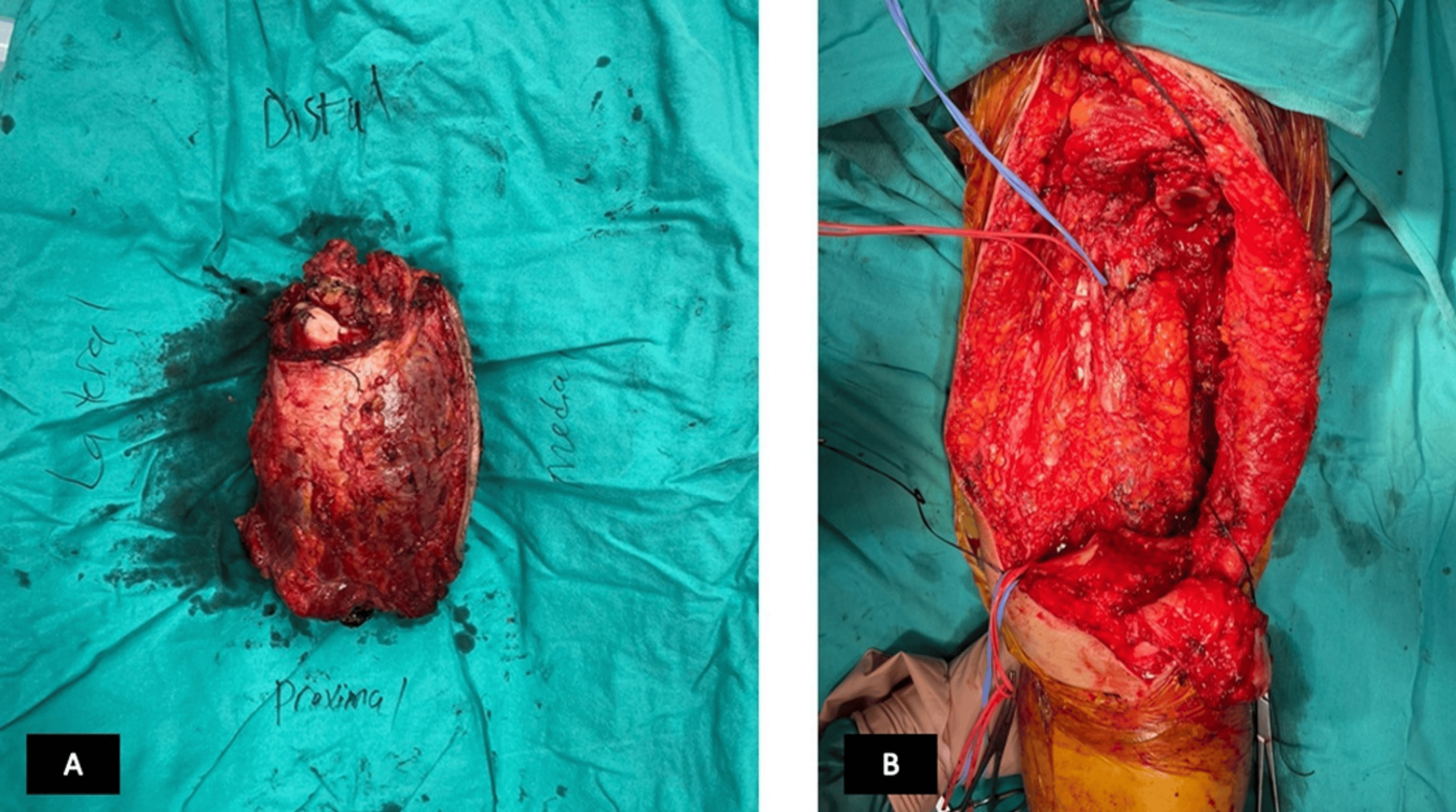
Peroperative images of the patient. (A) Lesion after removal. (B) The remaining tissues after removal.

The resection materials were sent for pathology, and intraoperative frozen section analysis yielded negative surgical margins, allowing the surgery to proceed (Figure 6).

**Figure 7:**
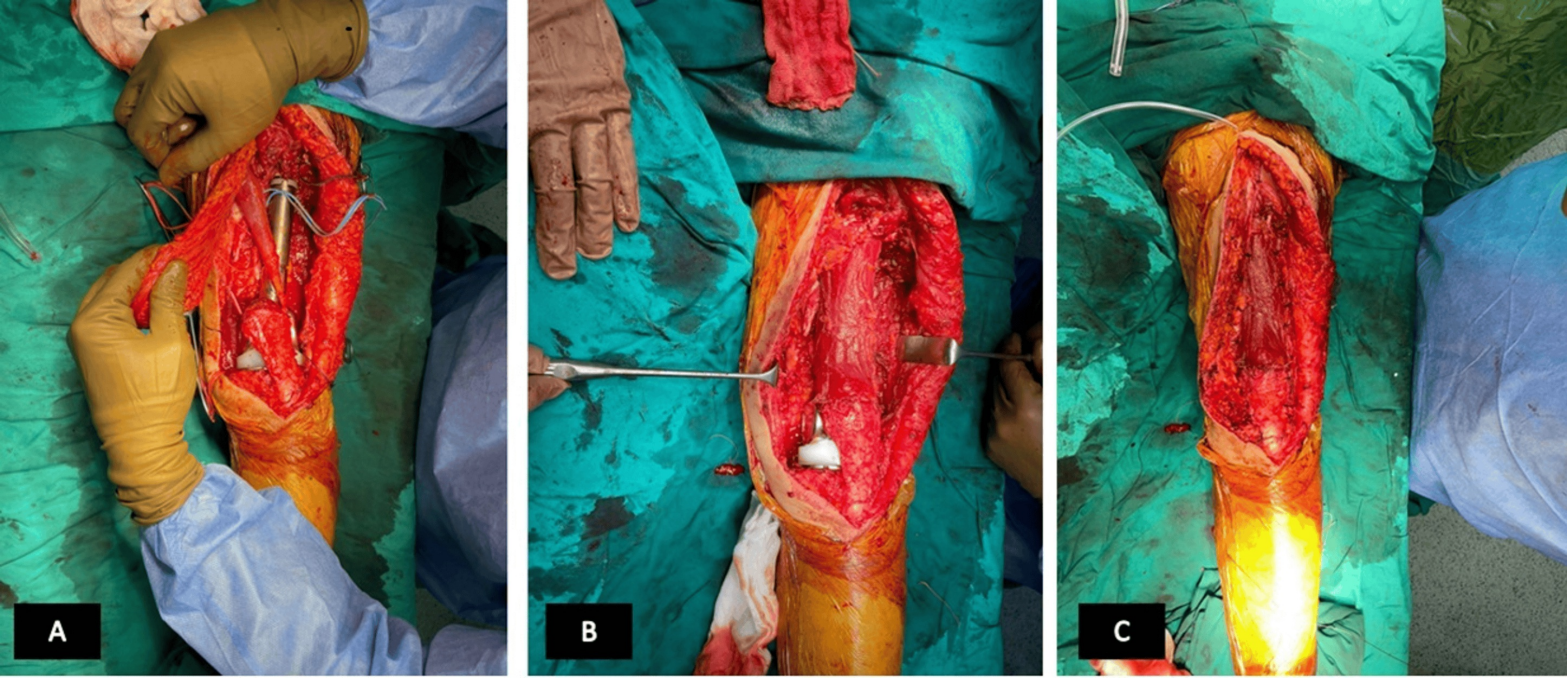
Peroperative images of the patient during the extensor mechanism reconstruction using sartorius and gracilis. (A) Anterior view of the prosthesis before the reconstruction. (B-C) View of the prosthesis after the gracilis and sartorius reconstruction.

The gracilis and sartorius muscles were harvested to restore the extensor mechanism in place of the resected quadriceps muscle. Subsequently, the knee joint was opened using a medial parapatellar approach. The gracilis and sartorius muscles were fixed to the patella with a single bio-anchor to restore the extensor mechanism (Figure 7).

**Figure 8:**
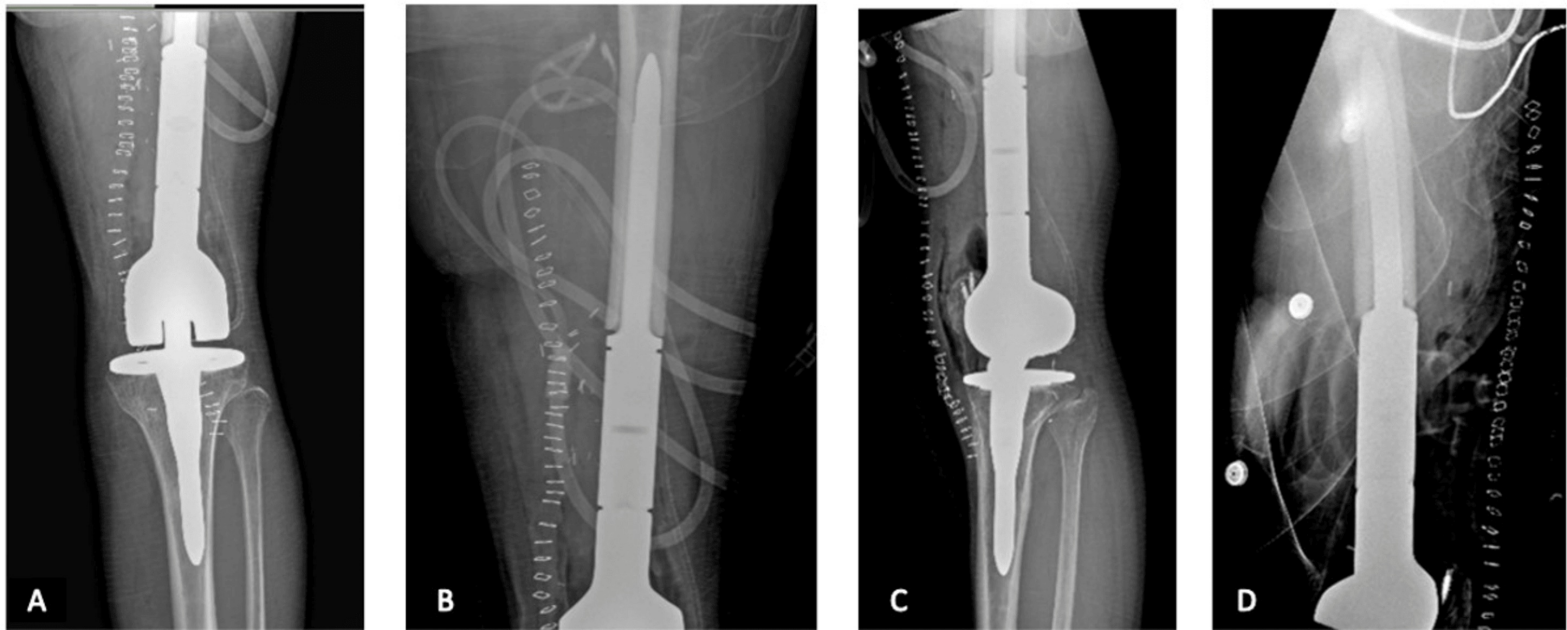
Postoperative X-ray images of the patient. (A-B) Anterior-posterior views of the left knee and femur respectively. (C-D) Lateral views of the left knee and femur respectively.

Intraoperatively, the range of motion assessment showed flexion up to 100 degrees. The patient was transferred to the intensive care unit postoperatively and then to the ward the following day without any complications at the surgical site. Follow-up X-rays taken after the surgery showed no pathological findings (Figure 8).

The early postoperative period proceeded smoothly without any loss of the extensor mechanism. Immunohistochemical analysis showed tumor cells negative for Desmin, patchy SMA positivity, and S100 positivity in macrophages. CD34 was negative in tumor cells but positive in vessels; ERG was positive in endothelial cells. Ki67 indicated high proliferation (50-60%). Resection revealed a 10.5×10×8 cm high-grade (Grade III) telangiectatic osteosarcoma with 10-20% necrosis, invading the joint surface, cortical bone, and soft tissues but sparing the synovial surface. No lymphovascular invasion was observed.

Following a multidisciplinary tumor board meeting, it was determined that chemotherapy would be the most appropriate course of treatment. Based on the evaluation by the radiation oncology department, radiotherapy was not recommended. The chemotherapy regimen administered consisted of cisplatin 140 mg and doxorubicin 120 mg. At the patient's one-year postoperative follow-up, a whole-body PET scan was performed, and no metastases were detected. The extensor mechanism was observed to be unchanged compared to the postoperative period.

## Discussion

Malignant mesenchymal tumors, originating from connective tissues, are aggressive cancers that affect various parts of the body. Treatment requires a personalized approach, considering the tumor type, location, stage, and patient health, typically involving a multidisciplinary strategy combining surgery, chemotherapy, and radiation therapy. The complexity of these tumors' origins involves a combination of genetic, environmental, and lifestyle factors. Specific genetic mutations and chromosomal irregularities contribute to their development, often triggered by exposure to carcinogens or genetic syndromes. These tumors frequently exhibit mutations in genes regulating cell growth, along with chromosomal abnormalities, which disrupt vital signaling pathways. Additionally, epigenetic modifications contribute to gene expression dysregulation in these tumors. We also confirmed the absence of distal metastases in our patient by conducting a PET-CT scan. Following detailed discussions in the tumor board, we planned wide resection and reconstruction with a tumor resection prosthesis for the patient [7].

Surgical intervention plays a vital role in managing malignant mesenchymal tumors (MMTs), with the primary objective of achieving complete tumor removal while preserving surrounding healthy tissues. Previous experiences with surgical removal of MMTs have demonstrated varying outcomes. Successful cases underscore the significance of wide excision with negative margins, which greatly reduces the risk of local recurrence and improves survival rates [8]. However, complications such as infection, local recurrence, or functional impairment have been reported, particularly in cases involving incomplete resections or tumors located in anatomically challenging regions. Factors influencing surgical outcomes include patient-specific factors, such as age, overall health, and tumor characteristics, as well as methodological factors, such as the accuracy of preoperative imaging, the surgeon's expertise, and the techniques employed during the procedure. In our case, we performed a wide resection and reconstruction with a tumor resection prosthesis, achieving clear margins. The patient was subsequently referred for adjuvant chemotherapy in the postoperative period to address potential residual microscopic disease and reduce the risk of recurrence, in line with best practices reported in the literature.

What distinguishes our case is that to prevent the loss of the extensor mechanism during surgery, we restored it by performing tendon transfers using the sartorius and gracilis muscles, which showed no tumor invasion, in place of the sacrificed quadriceps tendon.

As we had determined a very wide resection margin for the patient, preserving the quadriceps tendon did not seem feasible. Anticipating significant tissue loss, we requested the involvement of the plastic surgery team. However, upon considering the intact gracilis and sartorius muscles as potential compensation for the extensor mechanism, and with no significant soft tissue loss, we did not see the need for their intraoperative participation. Therefore, as the orthopedic team, we ensured the restoration of the extensor mechanism. During the patient's postoperative follow-ups, we did not encounter any loss of the extensor mechanism and achieved positive outcomes.

## Conclusions

In the treatment of malignant mesenchymal tumors, soft tissue loss is inevitable during wide resection and tumor resection prosthesis application. However, the use of the sartorius and gracilis tendons as patches to compensate for the loss of the extensor mechanism has not been demonstrated in the literature. It should be noted that such reconstructions can be attempted in cases where the quadriceps muscle cannot be preserved and is sacrificed.
